# Effects of dietary creatine supplementation on systemic microvascular density and reactivity in healthy young adults

**DOI:** 10.1186/1475-2891-13-115

**Published:** 2014-12-15

**Authors:** Roger de Moraes, Diogo Van Bavel, Beatriz Serpa de Moraes, Eduardo Tibiriçá

**Affiliations:** National Institute of Cardiology (INC), Rio de Janeiro, 21045-900 Brazil; Laboratory of Cardiovascular Investigation, Oswaldo Cruz Institute, Rio de Janeiro, Brazil; School of Physical Education and Sports Sciences of the Estácio de Sá University, Rio de Janeiro, Brazil

**Keywords:** Laser speckle contrast imaging, Intra-vital video-microscopy, Capillary recruitment, Post-occlusive reactive hyperemia

## Abstract

**Background:**

Dietary creatine supplementation (CrS) is a practice commonly adopted by physically active individuals. However, the effects of CrS on systemic microvascular reactivity and density have never been reported. Additionally, CrS is able to influence blood levels of homocysteine, resulting in presumed effects on vascular endothelial function. Thus, we investigated the effects of CrS on the systemic microcirculation and on homocysteine levels in healthy young individuals.

**Methods:**

This open-label study was performed on a group of 40 healthy male, moderately physically active subjects aged 27.7 ± 13.4 years who received one week of CrS at a dose of 20 g/day of commercially available micronized creatine monohydrate. Laser speckle contrast imaging was used in the evaluation of cutaneous microvascular reactivity, and intra-vital video microscopy was used to evaluate skin capillary density and reactivity, before and after CrS.

**Results:**

CrS did not alter plasma levels of homocysteine, although CrS increased creatinine (*p* = 0.0001) and decreased uric acid (*p* = 0.0004) plasma levels. Significant changes in total cholesterol (*p* = 0.0486) and LDL-cholesterol (*p* = 0.0027) were also observed along with a reduction in plasma levels of T3 (*p =* 0.0074) and an increase in T4 levels (*p* = 0.0003). Skin functional capillary density (*p* = 0.0496) and capillary recruitment during post-occlusive reactive hyperemia (*p* = 0.0043) increased after CrS. Increases in cutaneous microvascular vasodilation induced by post-occlusive reactive hyperemia (*p* = 0.0078) were also observed.

**Conclusions:**

Oral supplementation with creatine in healthy, moderately physically active young adults improves systemic endothelial-dependent microvascular reactivity and increases skin capillary density and recruitment. These effects are not concurrent with changes in plasma homocysteine levels.

## Introduction

Creatine supplementation (CrS) is a widely used practice implemented by athletes and physically active individuals with the goal of improving anaerobic power and to stimulate the process of protein synthesis and musculoskeletal hypertrophy [1].

CrS has antioxidant and cytoprotective activities [2] that, combined with the ability to restore intracellular energy levels, have also led to the introduction of this practice in therapies for the management of cardiovascular, neurologic, metabolic and muscle disorders [3–8].

In pathophysiological states wherein the intracellular levels of creatine are reduced, CrS has been shown to exert important neuromodulator action contributing to the treatment of anxiety disorders and schizophrenia and potentially to the prevention of Parkinson’s, Alzheimer’s and Huntington’s diseases [4].

Similarly, CrS has been used to treat muscular dystrophy and the idiopathic inflammatory myopathies in skeletal muscle diseases [3], to improve sarcolemma stabilization, arrhythmia frequency and contractile function in myocardium [7] and, in association with physical exercise, to increase glycemic control in patients with type 2 diabetes mellitus [9].

Nevertheless, few studies have investigated the direct effects of CrS on vascular function. In this context, it has been shown that creatine is capable of exerting anti-inflammatory actions on vascular endothelium [10] and lowering arterial stiffness evaluated after resistance exercise [11].

Considering that the synthesis of endogenous creatine is responsible for increasing hepatic demand on methylation reactions influencing homocysteine synthesis, it has been suggested that CrS is capable of reducing homocysteine blood levels, exerting positive influences on vascular endothelial function [12, 13]. Paradoxically, studies in humans suggest that CrS does not alter macrovascular reactivity but instead causes significant elevation of serum homocysteine in normohomocysteinemic subjects and reductions in hyperhomocysteinemic individuals [14, 15].

The assessment of systemic microvascular reactivity has already been proven to be essential in the investigation of the pathophysiology of cardiovascular and metabolic diseases [16]. Additionally, the cutaneous microcirculation is now considered as an accessible and representative vascular bed for the assessment of systemic microcirculatory reactivity and density [16, 17]. In this context, laser speckle contrast imaging (LSCI) provides an innovative approach for the non-invasive evaluation of systemic microvascular endothelial function [17, 18]. LSCI has already been shown to be an effective noninvasive technique in the evaluation of systemic microvascular reactivity in patients presenting with cardio-metabolic diseases [18]. Moreover, capillary density and reactivity, and thus tissue perfusion, are known to be closely correlated with cardiovascular and metabolic diseases, including arterial hypertension, diabetes, obesity and metabolic syndrome [19–21].

Given the absence of studies that elucidate the effects of CrS on systemic microvascular reactivity and density and to clarify the influences of this procedure on changes in plasma homocysteine levels, the present study aims to investigate the effect of CrS on the microcirculation and on homocysteine levels in healthy young individuals. Regarding the microcirculatory effects, we used LSCI coupled with physiological and pharmacological provocations in the evaluation of cutaneous microvascular reactivity and intra-vital video microscopy to evaluate skin capillary density and reactivity.

## Methods

### Subjects

This open-label study was performed on a group of 40 healthy male subjects aged 27.7 ± 13.4 years, recruited among the students of the School of Physical Education and Sports Sciences of the Estácio de Sá University, Rio de Janeiro, Brazil. The volunteers had negative family histories for cardiovascular and metabolic diseases, waist circumferences of 81.1 ± 12.0 cm and normal values for their lipid and glycemic profiles, according to the guidelines of the Brazilian Society of Cardiology (total cholesterol < 200 mg/dL; LDL-cholesterol < 160 mg/dL; triglycerides < 150 mg/dL and blood glucose < 100 mg/dL [22]). The study subjects were not highly trained and had not consumed any dietary supplement (creatine included) or medications for >3 months before the study; moreover, they were not instructed to follow a specific diet regimen. Even if the study subjects were not athletes, they were all physically active and were engaged in fitness programs involving aerobic activity and strength training at least three times a week. The present study was undertaken in accordance with the Helsinki declaration of 1975, as revised in 2000, and was approved by the Institutional Review Board (IRB) of the National Institute of Cardiology of Rio de Janeiro, Brazil under protocol number 53301, approved on September 2012. Once considered eligible, all of the subjects read and signed the informed consent form approved by the IRB.

### Research design

All evaluations were performed in the morning between 8 and 12 AM after a 12-hour fast. The subjects were also asked to refrain from smoking and to abstain from caffeine- and alcohol-containing beverages for 12 hours before the study. All procedures followed the same sequence, beginning with the collection of blood samples and followed by clinical and physical evaluation, concluding with the microcirculatory evaluation by LSCI and intra-vital capillaroscopy. The same procedures were repeated after one week of creatine supplementation.

Anthropometric evaluation consisted of measurements of weight, height and waist circumference (cm) and calculated body mass index (kg/m^2^). Systolic, diastolic and mean blood pressures were determined using a sphygmomanometer. The brachial systolic (SAP) and diastolic (DAP) blood pressures were measured twice, 1 minute apart, using a mercury sphygmomanometer, and the mean values were recorded as the patients’ clinical blood pressure. Mean arterial pressure (MAP) was calculated as DAP + 1/3 (SAP–DAP).

### Laboratory measurements

Blood specimens were collected before and after one week of creatine supplementation, and plasma samples were stored at -80°C until their utilization. Fasting glucose, total cholesterol, HDL cholesterol, triglycerides, creatinine, uric acid, transaminases, and high sensitivity CRP were determined by photometric colorimetric optical system (Cobas Mira systems, Roche Diagnostic Corporation, Indianapolis, IN, USA). LDL cholesterol was calculated by Friedewald’s formula. Plasma levels of homocysteine and fibrinogen were determined using an ELISA kit according to the manufacturer’s instructions (Cayman Chemical, Ann Arbor, MI, USA).

### Oral creatine supplementation

The subjects received 20 g/day of commercially available micronized creatine monohydrate with 99% purity by HPLC (Power Pure, Nutrisport, São Paulo, Brazil) for 1 week divided into 4 equal doses of 5 g, corresponding to the loading dose of the supplement according to previous reports [1, 23]. This study protocol has already been shown to significantly increase plasma and intramuscular levels of creatine without causing important side effects [23, 24].

### Evaluation of skin microvascular reactivity using laser speckle contrast imaging

Microcirculatory tests were performed after a 20-minute rest in the supine position in a temperature-controlled room (23 ± 1°C). Microvascular reactivity was evaluated using a laser speckle contrast imaging system with a laser wavelength of 785 nm (PeriCam PSI system, Perimed, Järfälla, Sweden) in combination with iontophoresis of acetylcholine (ACh) for noninvasive and continuous measurement of cutaneous microvascular perfusion changes (in arbitrary perfusion units, APU) [18, 25]. The image acquisition rate was 8 images/sec, and the distance between the laser head and the skin surface was fixed at 20 cm, as recommended by the manufacturer’s manual. Images were analyzed using the manufacturer’s software (PIMSoft, Perimed, Järfälla, Sweden). The skin sites for microvascular flow recordings were randomly chosen on the ventral surface of the forearm avoiding hair, broken skin, areas of skin pigmentation and visible veins. The drug-delivery electrode was secured using an adhesive disc (LI 611, Perimed, Järfälla, Sweden). Two measurement areas (circular regions of interest) of approximately 80 mm^2^ were determined. One of the measurement areas was within the electrode (acetylcholine), and the second (post-occlusive reactive hyperemia, PORH) was adjacent to the electrode. A vacuum cushion (AB Germa, Kristianstad, Sweden) was used to reduce recording artifacts generated by arm movements. ACh 2% w/v (Sigma Chemical CO, MO, USA) iontophoresis was performed using a micropharmacology system (PF 751 PeriIont USB Power Supply, Perimed, Sweden) with increasing anodal currents of 30, 60, 90, 120, 150 and 180 μA for 10-second intervals spaced 1 minute apart (the total charges were 0.3, 0.6, 0.9, 1.2, 1.5 and 1.8 mC, respectively). The dispersive electrode was attached approximately 15 cm away from the electrophoresis chamber. Of note, the drug was not injected but rather was placed in contact with the skin surface. During the PORH test, arterial occlusion was performed with suprasystolic pressure (50 mmHg above systolic arterial pressure) using a sphygmomanometer for 3 min. Following the release of pressure, the maximum flux was measured. Measurements of skin blood flow were divided by the mean arterial pressure to yield the cutaneous vascular conductance (CVC) in APU/mmHg. The amplitude of the PORH responses was expressed as the peak CVC minus the baseline CVC.

### Capillaroscopy by intra-vital microscopy

The microcirculatory tests were performed in an undisturbed quiet room with a defined stable temperature (23 ± 1°C) after a 20-minute rest in the supine position. The period of acclimatization lasted until the skin temperature had stabilized. We had previously shown that after 15-20 minutes of acclimatization, the skin temperature stabilizes at approximately 29°C [26].

The dorsum of the non-dominant middle phalanx was used for image acquisition, while the patient was maintained comfortably in a seated position. The room temperature was monitored and adjusted if necessary using air conditioning, considering that the outdoor temperature was usually > 25°C. The arm was positioned at the level of the heart and immobilized using a vacuum cushion (a specially constructed pillow filled with polyurethane foam that can be molded to any desired shape by creating a vacuum, from AB Germa, Kristianstad, Sweden).

Capillary density, i.e., the number of perfused capillaries per square millimeter of skin area, was assessed by high-resolution intra-vital color microscopy (Moritex, Cambridge, UK), as previously described and validated [19, 20, 26]. We used a video-microscopy system with an epi-illuminated fiberoptic microscope containing a 100-W mercury vapor lamp light source and an M200 objective with a final magnification of 200X. Images were acquired and saved for subsequent off-line analysis using a semi-automatic integrated system (Microvision Instruments, Evry, France). The mean capillary density for each patient was calculated as the arithmetic mean of visible (i.e., spontaneously perfused) capillaries in three contiguous microscopic fields of 1 mm^2^ each. For PORH, a blood pressure cuff was then applied around the patient’s arm and inflated to suprasystolic pressure (50 mm Hg greater than the systolic arterial pressure) to completely interrupt the blood flow for 3 minutes. This occlusion time has already been shown to effectively recruit capillaries in an endothelium-dependent manner [26]. After cuff release, images were again acquired and recorded over the subsequent 60-90 seconds, during which time the maximal hyperemic response was expected to occur.

The mean number of spontaneously perfused skin capillaries at rest is considered to represent the functional capillary density, as previously described [27]. Alternatively, the number of perfused capillaries during post-occlusive reactive hyperemia represents functional capillary recruitment, resulting from the release of endothelial mediators and consequent arteriolar vasodilation [27].

### Statistical analysis

The results were presented as the means ± SEM. For values that did not follow a Gaussian distribution, the medians (25th - 75th percentile) are presented (Shapiro-Wilk normality test). The results were analyzed using two-tailed paired Student’s *t* tests or Wilcoxon matched-pairs tests, respectively. *P* values <0.05 were considered statistically significant.

## Results

### Clinical, anthropometric and laboratory data

Table 1 shows the effects of creatine supplementation on the clinical and anthropometric data of the healthy volunteers. After one week of supplementation, an increase in total body mass (74.9 ± 1.8 vs. 75.4 ± 1.8 kg, *p* = 0.0020) and body mass index (25.2 ± 0.4 vs. 25.4 ± 0.5 kg/m^2^, *p* =0.0045) were observed along with a significant reduction in mean arterial pressure (92.1 ± 1.1 vs. 89.8 ± 1.1 mmHg, *p* = 0.0255). CrS did not alter plasma levels of homocysteine [10.5 (8.2-13.0) vs. 10.1 (8.8-12.3) μmol/L] but increased creatinine (0.92 ± 0.02 vs. 1.03 ± 0.03 mg/dL, *p* = 0.0001) and CK-MM [253 (146-567) vs. 344 (128-653) U/L, *p* = 0.0296] levels and decreased uric acid (4.9 ± 0.2 vs. 4.3 ± 0.2 mg/dL, *p* = 0.0004) plasma levels (Table 2). Fibrinogen levels were also decreased after CrS [282 (256-306) vs. 254 (227-284) mg/dL, *p* = 0.0177). The plasma lipid profile was also altered after CrS, with significant changes in total cholesterol [174.0 (143.5-204.0) vs. 174.0 (140.0-197.5) mg/dL, *p* = 0.0486] and LDL-C [115.0 (88.0-142.5) vs. 103 (81.0-130.0), *p* = 0.0027]. We also observed significant changes in total plasma proteins (7.3 ± 0.06 vs. 7.2 ± 0.07 g/dL, *p* = 0.0282) and globulins (3.1 ± 0.06 vs. 3.0 ± 0.08, *p* = 0.0588).

**Table 1:** **The clinical and anthropometric characteristics of the study subjects (n = 40) before and after one week of oral creatine supplementation**

Characteristics	Before creatine	After creatine	***p***value
Body mass (kg)	74.9 ± 1.8	75.4 ± 1.8	**0.0020**
Body mass index (kg/m^2^)	25.2 ± 0.4	25.4 ± 0.5	**0.0045**
Systolic blood pressure (mmHg)	124.7 ± 1.5	122.9 ± 1.5	0.1829
Diastolic blood pressure (mmHg)	75.6 ± 1.2	74.1 ± 1.4	0.3085
Mean blood pressure (mmHg)	92.1 ± 1.1	89.8 ± 1.1	**0.0255**
Heart rate (beats/min)	56.7 ± 1.5	57.5 ± 1.4	0.4904

The results were presented as the mean ± SEM.

*p* values were estimated using two-tailed paired Student’s *t* tests.

Bold values denote significant differences.

**Table 2:** **The laboratory characteristics of the study subjects (n = 40) before and after one week of oral creatine supplementation**

Characteristics	Before Creatine	After Creatine	***p***value
Homocysteine (μmol/L)	10.5 (8.2-13.0)	10.1 (8.8-12.3)	0.4434
Uric acid (mg/dL)	4.9 ± 0.2	4.3 ± 0.2	**0.0004**
Urea (mg/dL)	34.5 ± 1.5	35.7 ± 2.0	0.2482
Creatinine (mg/dL)	0.92 ± 0.02	1.03 ± 0.03	**0.0001**
CK-MB (U/L)	18.0 (11.5-25.0)	18.0 (13.0-27.0)	0.7601
CK-MM (U/L)	253 (146-567)	344 (128-653)	**0.0296**
Troponin (ng/mL)	0.0017 ± 0.0006	0.0029 ± 0.0010	0.4082
AST (U/L)	33.6 ± 1.8	36.4 ± 2.3	0.1718
ALT (U/L)	31.1 ± 2.6	30.1 ± 2.1	0.4380
Lactate dehydrogenase (U/L)	200.0 (159.5-335.0)	215.0 (166.0-315.5)	0.5694
Alkaline phosphatase (U/L)	59.0 (53.0-76.0)	58.0 (49.5-77.0)	0.5875
Fibrinogen (mg/dL)	282 (256-306)	254 (227-284)	**0.0177**
Triglycerides (mg/dL)	67.0 (56.6-91.5)	65.0 (51.5-91.0)	0.7420
Total cholesterol (mg/dL)	174.0 (143.5-204.0)	174.0 (140.0-197.5)	**0.0486**
HDL-C (mg/dL)	43.3 ± 1.8	44.6 ± 2.1	0.1999
LDL-C (mg/dL)	115.0 (88.0-142.5)	103 (81.0-130.0)	**0.0027**
Fasting glucose (mg/dL)	86.5 ± 1.0	86.0 ± 1.4	0.7298
Glycated hemoglobin (%)	5.3 ± 0.07	5.3 ± 0.09	0.7980
hs-CRP (mg/dL)	0.07 (0.04-0.19)	0.07 (0.04-0.18)	0.7645
Total protein (g/dL)	7.3 ± 0.06	7.2 ± 0.07	**0.0282**
Albumin (g/dL)	4.2 ± 0x.04	4.2 ± 0.04	0.6203
Globulins (g/dL)	3.1 ± 0.06	3.0 ± 0.08	**0.0588**
TSH (μUI/mL)	2.1 (1.4-3.0)	2.1 (1.5-2.7)	0.4788
T3 (ng/dL)	1.08 ± 0.03	1.02 ± 0.03	**0.0074**
T4 (ng/dL)	1.08 ± 0.02	1.1 ± 0.02	**0.0003**

The results are presented as the mean ± SEM. For values that did not follow a Gaussian distribution, the medians (25^th^ - 75^th^ percentile) are presented (Shapiro-Wilk normality test).

HDL-C: high-density lipoprotein cholesterol; LDL-C: low-density lipoprotein cholesterol, CK-MB: Creatine Kinase-MB; CK-MM: Creatine Kinase-MM; AST: Aspartate transaminase; ALT: Alanine transaminase; hs-CRP: high-sensitivity C-reactive protein; TSH: thyroid stimulating hormone; T3: triiodothyronine; T4: thyroxine.

*p* values were estimated using two-tailed unpaired Student’s *t* tests or Wilcoxon matched-pairs tests, as appropriate.

Bold values denote significant differences.

Finally, after CrS a reduction in plasma levels of T3 (1.08 ± 0.03 vs. 1.02 ± 0.03 ng/dL, *p =* 0.0074) and an increase in T4 levels (1.08 ± 0.02 vs. 1.1 ± 0.02 ng/dL, *p* = 0.0003) were observed.

### Microcirculatory parameters

#### Video-capillaroscopy

**Figure 1:**
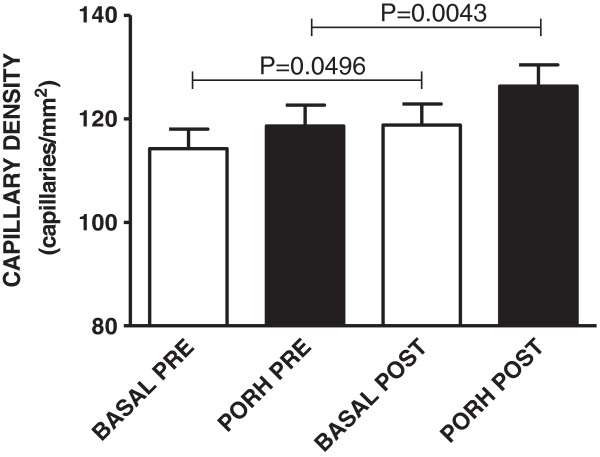
**Capillary density at baseline (BASAL) and during post-occlusive reactive hyperemia (PORH) of healthy young subjects (n = 40) before (PRE) and after (POST) oral creatine supplementation.** Values represent the mean ± SEM and were analyzed using two-tailed paired Student’s *t* tests.

Functional capillary density (basal capillary density) of the healthy volunteers was significantly increased after one week of CrS (114 ± 4 vs. 119 ± 4 capillaries/mm^2^, *p* = 0.0496). An increase in capillary recruitment during post-occlusive reactive hyperemia (119 ± 4 vs. 126 ± 4 capillaries/mm^2^, *p* = 0.0043) was also observed (Figure 1).

### Microvascular flow and reactivity

#### Microvascular responses to acetylcholine (Ach) stimulation

**Figure 2:**
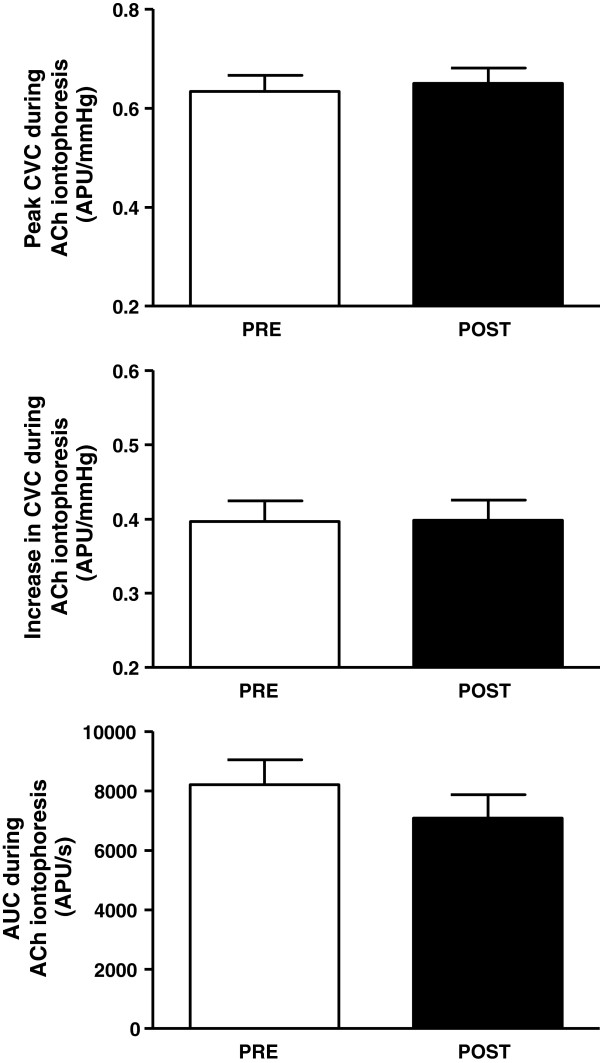
**The peak effects of skin iontophoresis of acetylcholine (ACh) on cutaneous microvascular conductance (CVC, expressed in arbitrary perfusion units, APU, divided by mean arterial pressure in mmHg, upper panel); increases in CVC induced by iontophoresis of ACh (middle panel) and the area under the curve (AUC) of skin iontophoresis of ACh (lower panel) of healthy young subjects (n = 40) before (PRE) and after (POST) oral creatine supplementation.** The amplitudes of ACh responses are expressed as peak CVC minus the baseline CVC. Values represent the means ± SEM.

One week of CrS did not alter microvascular vasodilation induced by skin iontophoresis of ACh (Figure 2). Peak values of cutaneous vascular conductance (CVC) were 0.63 ± 0.03 before and 0.65 ± 0.03 APU/mmHg after CrS; increases in CVC after ACh were 0.40 ± 0.03 vs. 0.40 ± 0.02 APU/mmHg and the area under the curve of ACh-induced vasodilation was 8212 ± 831 vs. 7089 ± 784 APU/s.

#### Microvascular responses to post-occlusive reactive hyperemia (PORH)

**Figure 3:**
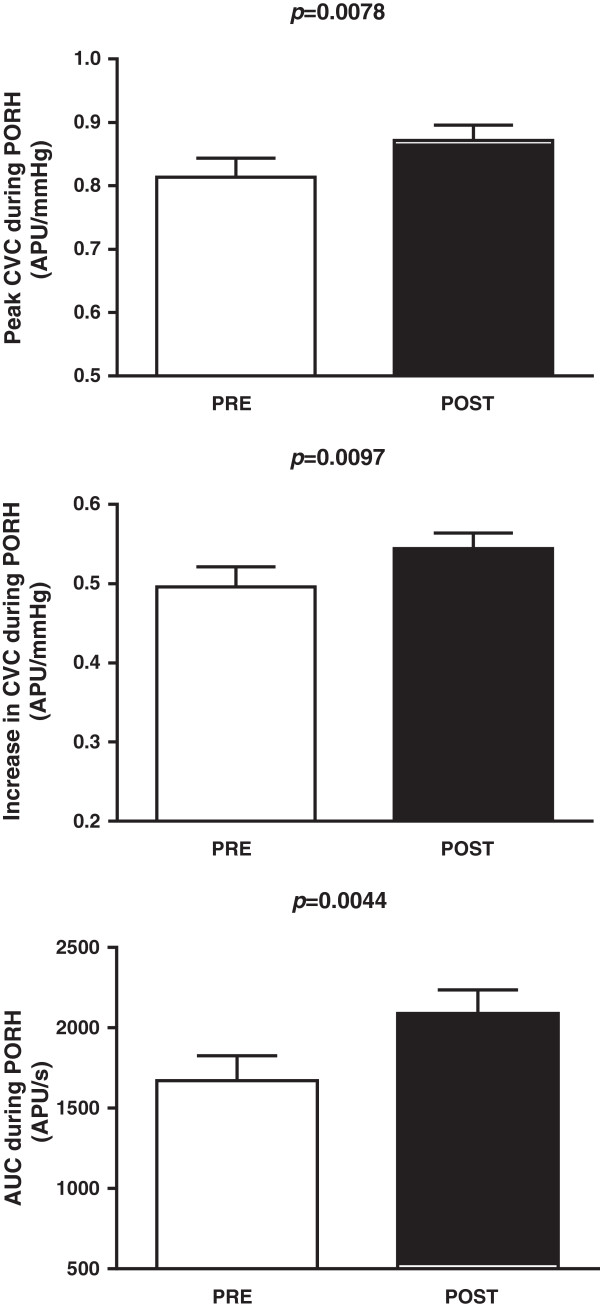
**The peak effects of post-occlusive reactive hyperemia (PORH) on cutaneous microvascular conductance (CVC, expressed in arbitrary perfusion units, APU, divided by mean arterial pressure in mmHg, upper panel); increases in CVC induced by PORH (middle panel) and the area under the curve (AUC) during PORH (lower panel) of healthy young subjects (n = 40) before (PRE) and after (POST) oral creatine supplementation.** The amplitudes of PORH responses are expressed as peak CVC minus the baseline CVC. Values represent the means ± SEM and were analyzed using two-tailed paired Student’s *t* tests.

After one week of CrS, we observed significant increases in microvascular vasodilation induced by PORH (Figure 3). Peak values of CVC were 0.81 ± 0.03 before and 0.87 ± 0.02 APU/mmHg after CrS (*p* = 0.0078); increases in CVC after PORH were 0.49 ± 0.02 vs. 0.54 ± 0.02 APU/mmHg (*p* = 0.0097) and the area under the curve of PORH-induced vasodilation was 1671 ± 146 vs. 2089 ± 146 APU/s (*p* = 0.0044).

## Discussion

The main findings of this study are as follows: i) oral supplementation with creatine monohydrate in healthy, moderately physically active young adults improves systemic endothelial-dependent microvascular reactivity; ii) the supplementation also increased skin capillary density and recruitment, which are dependent on microvascular endothelial function; and iii) blood pressure was also reduced after the supplementation.

The aforementioned changes occurred simultaneously with an increase in total body mass, most likely associated with fluid retention caused by the intracellular osmotic effect of creatine [6]. Similarly, we observed significant increases in creatinine and creatine kinase (MM fraction), and decreases in plasma levels of total proteins (caused by a decrease in globulins), uric acid, total cholesterol and LDL-cholesterol.

Our results also demonstrated that, unlike the results of previous studies [13, 14], CrS neither reduced nor increased serum homocysteine levels. In this regard, it should be emphasized that our sample involved young and physically active individuals, justifying further investigation to elucidate the influence of CrS on plasma levels of homocysteine among patients with cardio-metabolic diseases. Creatine supplementation is primarily indicated in athletes; nevertheless, it is widespread practice to use nutritional supplements (including creatine) to potentiate the effects of exercise training in the alterations of body composition [16]. In this context, the protocol of creatine supplementation in a dose of 20g/day during 5-7 days, followed by a dose of 5g/day during 20-30 days, has been shown to increase significantly creatine levels in skeletal muscle and eventually to improve strength gain and muscular hypertrophy in non-athletes but physically active individuals [1, 16–19] Considering that most studies evaluating the effects of creatine supplementation on plasma homocysteine levels have presented conflicting results, we decided to start our studies of creatine supplementation in young, physically active healthy subjects before using it in patients, mainly for security reasons. As a second step, we intend to test the effects of creatine supplementation in patients presenting with diabetes, hypertension and dyslipidemia, with and without hyperhomocysteinemia in future studies.

Even if CrS did not alter microvascular acetylcholine-mediated dilation, it significantly increased microvascular flow after post-occlusive reactive hyperemia (PORH). In this context, it has been suggested that although the response to acetylcholine-mediated dilatation is largely dependent on nitric oxide, those mediated by reactive hyperemia, at least in the skin microcirculation, occur independently of this endogenous mediator [28]. According to Cracowski and colleagues [29], the cutaneous microvascular flow-mediated dilatation of healthy individuals is predominantly dependent on sensory nerves and epoxygenase metabolites, particularly epoxyeicosatrienoic acid (EET), most likely related to the endothelium-derived hyperpolarization factor (EDHF), which might influence the activation of calcium-dependent potassium channels in vascular smooth muscle [30]. Although further studies are necessary regarding this issue, it is possible that CrS somehow contributes to increased EET bioavailability and may represent an important adjuvant therapy to improve endothelial function that is depressed in several metabolic and cardiovascular diseases.

Alternatively, vasodilation of the cutaneous microcirculation observed during reactive hyperemia might have been mediated by ATP-dependent potassium channels’ (K^+^_ATP_) activation in the endothelium and smooth muscle of the arterioles [31, 32]. In fact, evidence exists for the presence of the enzyme creatine kinase functionally coupled to the K^+^_ATP_ channels [33] that could be activated by eicosanoids such as EET or by low cellular energy signals [34]. Thus, it is possible that the increased intracellular creatine levels in tissues such as the endothelium are able to activate K^+^_ATP_ channels, hyperpolarize the vascular smooth muscle, and contribute to the enhancement in hyperemia-mediated dilatation found in our study.

Moreover, Prass and colleagues [35] proposed that creatine may exert a direct vascular action and is involved in the potentiation of the reactive hyperemia response after ischemia in stroke experimental models, allowing a more rapid recovery in these animals. Because the existence of the creatine transporter is well established [36] as well as the presence of large phosphocreatine reserves in vascular endothelium [37], and their sensitivity is believed to be increased through exogenous supplementation [35], it is reasonable to speculate that the creatine supplementation was involved in the alterations of microvascular reactivity observed in our study. Notwithstanding, independent of the mediators involved in flow-mediated microvascular vasodilation, our results indicate an improvement of microvascular endothelial function after creatine supplementation.

It has also been suggested that CrS is able to signal an intracellular energy deficit because it induces significant increases in the creatine kinase-phosphocreatine ratio (Cr/PCr ratio) in skeletal muscle [38]. Consistent with this hypothesis, it has been shown that CrS can increase mitochondrial oxidative phosphorylation [39] as well as glucose oxidation in skeletal muscle [38, 40] and to stimulate 5' AMP-activated protein kinase (AMPK) [38, 41], contributing to cellular adaptations that enhance energy production. In this context, it is possible that increases in intracellular creatine concentration, particularly in skeletal muscle, where creatine is mostly stored, contributed to the total and LDL-cholesterol serum reductions observed after a week of CrS. In fact, it has been demonstrated that CrS is able to improve the lipid profile in humans and may play a role in supporting physical training as a therapy in hypercholesterolemic individuals, an effect most likely associated with the capacity of creatine to activate the Krebs cycle and oxidative phosphorylation [42].

Interestingly, our results showed that CrS reduces tissue conversion of T4 to T3, which occurs predominantly in the kidneys and skeletal muscle through the action of the type 2 deiodinase enzyme [43]. Because the conversion of T4 to T3 requires energy and considering that increases in the Cr/PCr ratio signals tissue energy depletion, it is possible that changes in the plasma levels of thyroid hormones resulted from the CrS. Because glucose transporter type 4 (GLUT-4) synthesis is T3 dependent, this result would explain why CrS was not able to increase intramuscular glucose uptake, even if it might have activated AMPK, as demonstrated in a previous study [38]. In this regard, there is evidence that creatine increases membrane GLUT-4 translocation in skeletal muscle fibers [9].

Although energy overload can increase T3 availability [44], low energy levels represented by a high Cr/PCr ratio might signal the reduction of type 2 deiodinase activity in the kidneys and skeletal muscle, leading to a reduced conversion of T4 to T3 in those tissues [45]. Alternatively, because intramuscular creatine transport is an ATP-dependent process [46], it is possible that the increases in the intracellular creatine flow might have reduced ATP availability for T4 transport.

The conceivable reductions in type 2 deiodinase activity and T3 levels in skeletal muscle might have contributed to the elevated serum CK levels [47] observed in our study after a week of CrS. Alternatively, the increases in intracellular osmolarity produced by CrS might have contributed to muscle fiber disruption and CK release into the blood [1]. In this sense, it has been clearly demonstrated that reductions in plasma levels of T3, occurring in clinical and subclinical hypothyroidism, affects skeletal muscle, increasing membrane permeability to CK and thus resulting in increases of the plasma concentrations of the enzyme [48, 49].

Even if the exposure of the ventricular myocardium to T3 reduces the amount of membrane Na^+^/Cr transporter mRNA [50], it has also been proposed that the exposure of muscle cells to this hormone could increase Na^+^/K^+^-ATPase activity because increases in extracellular Na^+^ concentrations would positively influence creatine transport within muscle fibers [51]. It is also possible that the T3 reduction found in our study represents a mechanism that acts to limit creatine transport that might produce irreversible cellular osmotic damage. Thus, it is possible that excessive increases in creatine supply contributes to a compensatory reduction in T3 synthesis by decreasing the activity of the type 2 deiodinase in tissues such as the kidneys and skeletal muscle, explaining increased plasma CK levels [52].

In our study, a week of CrS significantly increased creatinine and CK plasma levels, and simultaneously reduced globulins and T3 plasma levels, mimicking a condition that characterizes impaired renal function [53]. Understanding that CrS may contribute to renal dysfunction misdiagnosis because moderate increases of creatinine levels are to be expected [54, 55], evidence indicates that creatine supplementation would overload kidney function [1, 56]. Although several studies ensure the safety of CrS [57–61], even in individuals at risk for kidney disease, daily doses of 20 g were associated with the formation of carcinogenic heterocyclic amines and to deleterious molecules such as methylamine and formaldehyde that promote cross-linkage between proteins and DNA damage-induced changes to renal structures [62, 63]. Because most studies that have attested to CrS safety were performed in association with physical exercise, it is possible that the deleterious effects on renal function are observed only among individuals who are not enrolled in well-controlled exercise training programs, as was the case in our sample. In fact, it has been shown that CrS in rats produces deleterious renal effects in sedentary animals but is safe in those maintained on regular physical training [64].

### Limitations and strengths of the study

One important limitation of the present study is the lack of a placebo-controlled double-blind supplementation methodology. Notwithstanding, our study included a fairly high number of healthy volunteers (n = 40), yielding very reproducible results, demonstrated by the rather low dispersion of the values of metabolic and microcirculatory variables. Moreover, it has already been clearly demonstrated that the reproducibility of laser speckle contrast imaging methodology in the evaluation of skin microvascular reactivity is very high [17, 65–67].

Another limitation concerning the conclusions of the study could be the marginally statistically significant changes in thyroid hormones and microvascular reactivity. It is conceivable that these alterations might not be clinically relevant in healthy young adults, since they do not have microvascular endothelial dysfunction, as previously demonstrated by our group using laser speckle contrast imaging [18]. Nevertheless, these modest but statistically significant improvements of microvascular function observed in our study after creatine supplementation in healthy volunteers could turn out to be clinically relevant in patients with cardiovascular and metabolic diseases. Moreover, even small alterations of plasma concentrations of the thyroid hormones indicate that creatine supplementation might influence thyroid metabolism. Considering the widespread use of creatine supplementation by athletes and also by non-athletes in fitness centers, one must be cautious in the association of the creatine supplementation with drugs that potentially interfere with thyroid metabolism such as drugs acting in the central nervous system (carbamazepine, lithium) and steroid hormones (glucocorticoids) [68, 69].

In conclusion, oral supplementation with creatine monohydrate in healthy, moderately physically active young adults improves systemic endothelial-dependent microvascular reactivity and increases skin capillary density and recruitment. These effects are not concurrent with changes in the plasma levels of homocysteine.

## Abbreviations

ACh: Acetylcholine
APU: Arbitrary perfusion units
AUC: Area under the curve
CVC: Cutaneous vascular conductance
CrS: Creatine supplementation
Cr/PCr ratio: Creatine kinase-phosphocreatine ratio
CK: Creatine kinase
EET: Epoxyeicosatrienoic acid
EDHF: Endothelium-derived hyperpolarization factor
LSCI: Laser speckle contrast imaging
PORH: Post-occlusive reactive hyperemia.

## References

[1] Hall M, Trojian TH (2013). Creatine supplementation. Curr Sports Med Rep.

[2] Sestili P, Martinelli C, Bravi G, Piccoli G, Curci R, Battistelli M, Falcieri E, Agostini D, Gioacchini AM, Stocchi V (2006). Creatine supplementation affords cytoprotection in oxidatively injured cultured mammalian cells via direct antioxidant activity. Free Radic Biol Med.

[3] Kley RA, Tarnopolsky MA, Vorgerd M (2013). Creatine for treating muscle disorders. Cochrane Database Syst Rev.

[4] Allen PJ (2012). Creatine metabolism and psychiatric disorders: Does creatine supplementation have therapeutic value?. Neurosci Biobehav Rev.

[5] Gualano B, Roschel H, Lancha-Jr AH, Brightbill CE, Rawson ES (2012). In sickness and in health: the widespread application of creatine supplementation. Amino Acids.

[6] Adhihetty PJ, Beal MF (2008). Creatine and its potential therapeutic value for targeting cellular energy impairment in neurodegenerative diseases. Neuromolecular Med.

[7] Strumia E, Pelliccia F, D'Ambrosio G (2012). Creatine phosphate: pharmacological and clinical perspectives. Adv Ther.

[8] Persky AM, Brazeau GA (2001). Clinical pharmacology of the dietary supplement creatine monohydrate. Pharmacol Rev.

[9] Gualano B, DE Salles Painneli V, Roschel H, Artioli GG, Neves M, De Sa Pinto AL, Da Silva ME, Cunha MR, Otaduy MC, Leite Cda C, Ferreira JC, Pereira RM, Brum PC, Bonfá E, Lancha AH (2011). Creatine in type 2 diabetes: a randomized, double-blind, placebo-controlled trial. Med Sci Sports Exerc.

[10] Nomura A, Zhang M, Sakamoto T, Ishii Y, Morishima Y, Mochizuki M, Kimura T, Uchida Y, Sekizawa K (2003). Anti-inflammatory activity of creatine supplementation in endothelial cells in vitro. Br J Pharmacol.

[11] Sanchez-Gonzalez MA, Wieder R, Kim JS, Vicil F, Figueroa A (2011). Creatine supplementation attenuates hemodynamic and arterial stiffness responses following an acute bout of isokinetic exercise. Eur J Appl Physiol.

[12] Deminice R, Portari GV, Vannucchi H, Jordao AA (2009). Effects of creatine supplementation on homocysteine levels and lipid peroxidation in rats. Br J Nutr.

[13] McCarty MF (2001). Supplemental creatine may decrease serum homocysteine and abolish the homocysteine 'gender gap' by suppressing endogenous creatine synthesis. Med Hypotheses.

[14] Jahangir E, Vita JA, Handy D, Holbrook M, Palmisano J, Beal R, Loscalzo J, Eberhardt RT (2009). The effect of L-arginine and creatine on vascular function and homocysteine metabolism. Vasc Med.

[15] Deminice R, Rosa FT, Franco GS, da Cunha SF, de Freitas EC, Jordao AA (2013). Short-term creatine supplementation does not reduce increased homocysteine concentration induced by acute exercise in humans. Eur J Nutr.

[16] Holowatz LA, Thompson-Torgerson CS, Kenney WL (2008). The human cutaneous circulation as a model of generalized microvascular function. J Appl Physiol.

[17] Roustit M, Cracowski JL (2013). Assessment of endothelial and neurovascular function in human skin microcirculation. Trends Pharmacol Sci.

[18] Cordovil I, Huguenin G, Rosa G, Bello A, Kohler O, de Moraes R, Tibirica E (2012). Evaluation of systemic microvascular endothelial function using laser speckle contrast imaging. Microvasc Res.

[19] Debbabi H, Uzan L, Mourad JJ, Safar M, Levy BI, Tibirica E (2006). Increased skin capillary density in treated essential hypertensive patients. Am J Hypertens.

[20] Kaiser SE, Sanjuliani AF, Estato V, Gomes MB, Tibirica E (2013). Antihypertensive treatment improves microvascular rarefaction and reactivity in low-risk hypertensive individuals. Microcirculation.

[21] Serne EH, de Jongh RT, Eringa EC, IJzerman RJ, Stehouwer CD (2007). Microvascular dysfunction: a potential pathophysiological role in the metabolic syndrome. Hypertension.

[22] Sposito AC, Caramelli B, Fonseca FA, Bertolami MC, Afiune Neto A, Souza AD, Lottenberg AM, Chacra AP, Faludi AA, Loures-Vale AA, Carvalho AC, Duncan B, Gelonese B, Polanczyk C, Rodrigues Sobrinho CR, Scherr C, Karla C, Armaganijan D, Moriguchi E, Saraiva F, Pichetti G, Xavier HT, Chaves H, Borges JL, Diament J, Guimarães JI, Nicolau JC, dos Santos JE, de Lima JJ, Vieira JL (2007). [IV Brazilian Guideline for Dyslipidemia and Atherosclerosis prevention: Department of Atherosclerosis of Brazilian Society of Cardiology]. Arq Bras Cardiol.

[23] Jager R, Purpura M, Shao A, Inoue T, Kreider RB (2011). Analysis of the efficacy, safety, and regulatory status of novel forms of creatine. Amino Acids.

[24] Graham AS, Hatton RC (1999). Creatine: a review of efficacy and safety. J Am Pharm Assoc.

[25] Souza EG, De Lorenzo A, Huguenin G, Oliveira GM, Tibirica E (2014). Impairment of systemic microvascular endothelial and smooth muscle function in individuals with early-onset coronary artery disease: studies with laser speckle contrast imaging. Coron Artery Dis.

[26] Tibirica E, Rodrigues E, Cobas RA, Gomes MB (2007). Endothelial function in patients with type 1 diabetes evaluated by skin capillary recruitment. Microvasc Res.

[27] Antonios TF, Rattray FE, Singer DR, Markandu ND, Mortimer PS, MacGregor GA (1999). Maximization of skin capillaries during intravital video-microscopy in essential hypertension: comparison between venous congestion, reactive hyperaemia and core heat load tests. Clin Sci (Lond).

[28] Wong BJ, Wilkins BW, Holowatz LA, Minson CT (2003). Nitric oxide synthase inhibition does not alter the reactive hyperemic response in the cutaneous circulation. J Appl Physiol (1985).

[29] Cracowski JL, Gaillard-Bigot F, Cracowski C, Sors C, Roustit M, Millet C (2013). Involvement of cytochrome epoxygenase metabolites in cutaneous postocclusive hyperemia in humans. J Appl Physiol (1985).

[30] Lorenzo S, Minson CT (2007). Human cutaneous reactive hyperaemia: role of BKCa channels and sensory nerves. J Physiol.

[31] Wang H, Long C, Duan Z, Shi C, Jia G, Zhang Y (2007). A new ATP-sensitive potassium channel opener protects endothelial function in cultured aortic endothelial cells. Cardiovasc Res.

[32] Long CL, Qin XC, Pan ZY, Chen K, Zhang YF, Cui WY, Liu GS, Wang H (2008). Activation of ATP-sensitive potassium channels protects vascular endothelial cells from hypertension and renal injury induced by hyperuricemia. J Hypertens.

[33] Selivanov VA, Alekseev AE, Hodgson DM, Dzeja PP, Terzic A (2004). Nucleotide-gated KATP channels integrated with creatine and adenylate kinases: amplification, tuning and sensing of energetic signals in the compartmentalized cellular environment. Mol Cell Biochem.

[34] Shi WW, Yang Y, Shi Y, Jiang C (2012). K(ATP) channel action in vascular tone regulation: from genetics to diseases. Sheng li xue bao.

[35] Prass K, Royl G, Lindauer U, Freyer D, Megow D, Dirnagl U, Stockler-Ipsiroglu G, Wallimann T, Priller J (2007). Improved reperfusion and neuroprotection by creatine in a mouse model of stroke. J Cereb Blood Flow Metab.

[36] Braissant O (2012). Creatine and guanidinoacetate transport at blood-brain and blood-cerebrospinal fluid barriers. J Inherit Metab Dis.

[37] Decking UK, Alves C, Wallimann T, Wyss M, Schrader J (2001). Functional aspects of creatine kinase isoenzymes in endothelial cells. Am J Physiol Cell Physiol.

[38] Ceddia RB, Sweeney G (2004). Creatine supplementation increases glucose oxidation and AMPK phosphorylation and reduces lactate production in L6 rat skeletal muscle cells. J Physiol.

[39] Tonkonogi M, Harris B, Sahlin K (1998). Mitochondrial oxidative function in human saponin-skinned muscle fibres: effects of prolonged exercise. J Physiol.

[40] Eijnde BO, Derave W, Wojtaszewski JF, Richter EA, Hespel P (2005). AMP kinase expression and activity in human skeletal muscle: effects of immobilization, retraining, and creatine supplementation. J Appl Physiol (1985).

[41] Schoch RD, Willoughby D, Greenwood M (2006). The regulation and expression of the creatine transporter: a brief review of creatine supplementation in humans and animals. J Int Soc Sports Nutr.

[42] Gualano B, Ugrinowitsch C, Artioli GG, Benatti FB, Scagliusi FB, Harris RC, Lancha AH (2008). Does creatine supplementation improve the plasma lipid profile in healthy male subjects undergoing aerobic training?. J Int Soc Sports Nutr.

[43] Mullur R, Liu YY, Brent GA (2014). Thyroid hormone regulation of metabolism. Physiol Rev.

[44] Araujo RL, Andrade BM, Padron AS, Gaidhu MP, Perry RL, Carvalho DP, Ceddia RB (2010). High-fat diet increases thyrotropin and oxygen consumption without altering circulating 3,5,3'-triiodothyronine (T3) and thyroxine in rats: the role of iodothyronine deiodinases, reverse T3 production, and whole-body fat oxidation. Endocrinology.

[45] Araujo RL, Carvalho DP (2011). Bioenergetic impact of tissue-specific regulation of iodothyronine deiodinases during nutritional imbalance. J Bioenerg Biomembr.

[46] Snow RJ, Murphy RM (2001). Creatine and the creatine transporter: a review. Mol Cell Biochem.

[47] Ranka R, Mathur R (2003). Serum creatine phosphokinase in thyroid disorders. Indian J Clin Biochem.

[48] Hekimsoy Z, Oktem IK (2005). Serum creatine kinase levels in overt and subclinical hypothyroidism. Endocr Res.

[49] Gaede JT (1977). Serum enzyme alterations in hypothyroidism before and after treatment. J Am Geriatr Soc.

[50] Queiroz MS, Shao Y, Berkich DA, Lanoue KF, Ismail-Beigi F (2002). Thyroid hormone regulation of cardiac bioenergetics: role of intracellular creatine. Am J Physiol Heart Circ Physiol.

[51] Odoom JE, Kemp GJ, Radda GK (1996). The regulation of total creatine content in a myoblast cell line. Mol Cell Biochem.

[52] Kurahashi M, Kuroshima A (1976). Mechanism of thyroid-induced creatinuria in rat, with special reference to creatine synthesis in liver and creatine loss from skeletal muscle. Jpn J Physiol.

[53] Basu G, Mohapatra A (2012). Interactions between thyroid disorders and kidney disease. Indian J Endocrinol Metab.

[54] Willis J, Jones R, Nwokolo N, Levy J (2010). Protein and creatine supplements and misdiagnosis of kidney disease. BMJ.

[55] Pline KA, Smith CL (2005). The effect of creatine intake on renal function. Ann Pharmacother.

[56] Souza WM, Heck TG, Wronski EC, Ulbrich AZ, Boff E (2013). Effects of creatine supplementation on biomarkers of hepatic and renal function in young trained rats. Toxicol Mech Methods.

[57] Gualano B, Ugrinowitsch C, Novaes RB, Artioli GG, Shimizu MH, Seguro AC, Harris RC, Lancha AH (2008). Effects of creatine supplementation on renal function: a randomized, double-blind, placebo-controlled clinical trial. Eur J Appl Physiol.

[58] Lugaresi R, Leme M, de Salles PV, Murai IH, Roschel H, Sapienza MT, Lancha Junior AH, Gualano B (2013). Does long-term creatine supplementation impair kidney function in resistance-trained individuals consuming a high-protein diet?. J Int Soc Sports Nutr.

[59] Groeneveld GJ, Beijer C, Veldink JH, Kalmijn S, Wokke JH, van den Berg LH (2005). Few adverse effects of long-term creatine supplementation in a placebo-controlled trial. Int J Sports Med.

[60] Poortmans JR, Auquier H, Renaut V, Durussel A, Saugy M, Brisson GR (1997). Effect of short-term creatine supplementation on renal responses in men. Eur J Appl Physiol Occup Physiol.

[61] Poortmans JR, Francaux M (2000). Adverse effects of creatine supplementation: fact or fiction?. Sports Med.

[62] Kim HJ, Kim CK, Carpentier A, Poortmans JR (2011). Studies on the safety of creatine supplementation. Amino Acids.

[63] Yu PH, Deng Y (2000). Potential cytotoxic effect of chronic administration of creatine, a nutrition supplement to augment athletic performance. Med Hypotheses.

[64] Souza RA, Miranda H, Xavier M, Lazo-Osorio RA, Gouvea HA, Cogo JC, Vieira RP, Ribeiro W (2009). Effects of high-dose creatine supplementation on kidney and liver responses in sedentary and exercised rats. J Sports Sci Med.

[65] Roustit M, Millet C, Blaise S, Dufournet B, Cracowski JL (2010). Excellent reproducibility of laser speckle contrast imaging to assess skin microvascular reactivity. Microvasc Res.

[66] Roustit M, Cracowski JL (2012). Non-invasive assessment of skin microvascular function in humans: an insight into methods. Microcirculation.

[67] Humeau-Heurtier A, Guerreschi E, Abraham P, Mahe G (2013). Relevance of laser Doppler and laser speckle techniques for assessing vascular function: state of the art and future trends. IEEE Trans Biomed Eng.

[68] Baumgartner A, Pinna G, Hiedra L, Gaio U, Hessenius C, Campos-Barros A, Eravci M, Prengel H, Thoma R, Meinhold H (1997). Effects of lithium and carbamazepine on thyroid hormone metabolism in rat brain. Neuropsychopharmacology.

[69] Dong BJ (2000). How medications affect thyroid function. West J Med.

